# Lessons learned about ageing and gerontological nursing in South Africa

**DOI:** 10.4102/curationis.v38i1.1216

**Published:** 2015-07-30

**Authors:** Staja Q. Booker

**Affiliations:** 1 The University of Iowa, College of Nursing, United States

## Abstract

**Background:**

The unprecedented global growth in older adults merits high-quality gerontological nursing care. As gerontological nursing grows in visibility in developed and developing countries, nurses must possess a broader worldview of ageing with knowledge of physiological, psychosocial, and cultural issues.

**Purpose:**

The purpose of this article is to: (1) highlight lessons learned on differences and similarities in ageing and care of older adults in the United States of America (USA) and South Africa (SA); and (2) provide recommendations on how to advance gerontological nursing education in SA.

**Methods:**

A two-week international service-learning project was undertaken by visiting SA and learning about their nursing system and care of older adults. Service-learning is an innovative teaching-learning-service method that provided reflective and hands-on experience of gerontological nursing. This article provides a personal reflection of lessons learned about ageing and gerontological nursing during the service-learning project.

**Findings:**

Care of older adults in SA is in many ways different from and similar to that in the USA. Consequently global nurses should recognise those differences and provide culturally appropriate care. This service-learning experience also demonstrated the need for gerontological nursing education in SA. Based on this, recommendations on how to infuse and advance gerontological nursing education in SA are provided.

**Conclusion:**

Caring for older adults in a global context requires knowledge and understanding of cultures and their values and practices. With a growing population of diverse older adults, there is a need for incorporation of more gerontological education in nursing curriculums and clinical experiences.

## Introduction

The global landscape for the care of older adults is changing in response to an ageing demographic. It is estimated that the global population of those aged 65 years and older will grow to 1.53 billion by 2050 (United States Census Bureau 2009). As of 2009 South Africa (SA) ranked 108th in size of older adult population, with 7% aged 60 years and older (United Nations 2009). In response to rising numbers of ageing adults, the SA Government passed the *Older Persons Act* in 2006, which aims to improve and maintain the health, safety and security of older persons (Republic of South Africa 2006). The gerontological perspective on the current state of practice, policy and education is emerging, as the International Association of Gerontology and Geriatrics (IAGG) held its first African regional conference in 2012 (IAGG 2012:11).

In response to an increase in older adults worldwide, nurses must be educationally prepared to care for this population, and this entails nurses and nursing students being able to recognise differences in cultural needs. Whilst there are similarities in care of older adults worldwide, there are unique considerations for SA elders.

According to Bohman, Van Wyk and Ekman (2010:194) a ‘…westernised individualistic perspective is not always applicable in a different cultural context’. A dearth of nursing research and practice has been published on eldercare in SA, and Bohman et al. (2010:187) agree that the welfare of older South Africans has not been given adequate attention. After being immersed in a two-week service-learning trip to SA, Bohman and colleagues’ conclusion became quite evident, and I was compelled to share my insight on ageing in SA and how gerontological nursing could be enhanced in SA.

Therefore, the purpose of this article is to share five lessons that were learned about ageing and gerontological nursing (aged care nursing) whilst in SA, and also to discuss areas for advancement of gerontological nursing education in SA in order to expand and improve care for elders.

## Methods

Service-learning is one method used to enhance the knowledge and practice of intergenerational gerontology and geriatrics (Karasik 2013). Because international service-learning is an effective tool for teaching principles on cross-cultural gerontology (Karasik 2013), many American nursing curricula include some aspect of service-learning, whether local, national or international (Amerson 2014:175). International nursing service-learning allows individuals to visit international countries and provide voluntary service whilst also participating in formal and/or informal learning experiences (Amerson 2014:177–178). Thus students gain a rare look at social determinants and healthcare practices, outcomes and policy issues in diverse populations. International service-learning is generally arranged and completed through collaboration of academic or service organisations.

In spring 2012 collaboration between a College of Nursing at a north-eastern university in the USA and a School of Nursing in SA allowed a group of American nursing professionals, undergraduate nursing students, and one graduate nursing student to embark on a service-learning experience in SA. A two-week international service-learning project was undertaken by visiting SA to learn about the nursing system and care of older adults in this country. The accompanying nursing graduate student (who is a geriatric nurse) used this service-learning project as an independent study course for completion of the adult health and/or gerontology nursing master's programme.

As told from the personal experience of the nursing graduate student (i.e. the author of this article), this article recounts five anecdotal lessons that were learned about ageing and gerontological nursing in SA. These lessons were learned through observation and casual conversations with nursing students, faculty, and practising nurses. No Institutional Review Board approval was needed for this trip.

## Findings

### Lesson 1: Definition of ‘older adult’ varies from country to country

‘Age is just a number’ is a common statement in the USA. Even some older South Africans denoted ‘being old’ or an ‘old person’ as someone possessing knowledge and wisdom, as opposed to someone of a certain age (Bohman et al. 2010:190). Given that 25 years is the median age of South Africans (United Nations 2011), the end of adulthood is then closer to 50 years of age. Therefore a person in their early to mid-fifties may be considered an ‘older adult’ in SA, given that the average life-expectancy is 55 years for males and 62 years for females (World Health Organization 2012).

This is distinctly different from the western view that persons of 65 years and older are considered older adults. I also learned that physiologically, persons with end-stage AIDS may resemble a ‘traditional’ older adult despite their chronological age; that is, they may display geriatric syndromes related to illness and polypharmacy.

Gaining such knowledge, it was concluded that no one definition of ‘older adult’ can be applied globally. Instead a definition should accurately identify the chronological and physiological needs of a country's population. King (2008:109–110) posits that more research is needed to clarify definitions of ageing for African cultures.

### Lesson 2: Environment greatly influences ageing in SA

Environmental issues greatly influence the ageing trajectory not only in SA but worldwide. The ecological environment of older persons can either hinder or facilitate successful, safe ageing. The concept of person-environment fit (P-E fit) emphasises the role of the physical environment, personal environment, small group environment, and social environment on the safety and match of older adults (Pomeroy et al. 2011). From my observations it was clear that numerous environmental issues, such as home safety, safe and healthy nutrition, violence, rapid urbanisation, and access to health care, impact all South Africans, including the older persons’ P-E fit.

Ensuring environmental safety is a foundation of gerontological nursing care. During my visit I observed that living conditions in the townships are very unsafe, as many of the homes are constructed of a combination of discarded and highly flammable materials. I learned of a very innovative campaign that seeks to reduce the number of candle-related fires in townships. Developed by a retired South African nurse this initiative, called ‘A Candle in a Jar’, and consists of a glass jar half-filled with sand in which a candle is placed; if the jar is accidently knocked over, the sand will extinguish the flame.

Healthy nutrition is another important concern in older adults, since they are prone to develop nutritional deficiencies as a consequence of ageing and lack of finance. Nutrition in an older adult living with HIV or AIDS is even more of a priority (Raiten et al. 2011). In order to promote healthy nutrition, community workers partner with a local university school of nursing, where they actively assist community residents to plant gardens as a means of producing sustainable and healthy food sources. Implementation of models for community partnerships will be integral to enhancing the health of communities and older adults in SA (Mtshali 2009; Koon, Goudge & Norris 2013; anecdotal observations).

### Lesson 3: Home-based care preferred over institutional care

There is an emerging ideological conflict between maintaining the tradition of home-based care versus embracing a transition to long-term institutional care (i.e. nursing homes or aged care homes, frail care homes, etc.). As with most African and African-descent ethnicities, caring for family in the home is regarded as a moral imperative and familial responsibility. This strong familial responsibility is even seen in African-Americans in the USA (Epps 2013:130). A traditional mainstay in African culture is enactment of intergenerational reciprocal care (Bohman, Van Wyk & Ekman 2009), which is an obligation and sign of respect (Bohman et al. 2010).

An ethnographic study revealed that some South Africans want to stay in-home, and ‘… don’t want to go to an old-age home’, whereas others view old-age homes as a real alternative (Bohman et al. 2009:452). Although there are old-age homes (i.e. nursing homes) and frail care services available in SA, intergenerational caregiving is at the forefront of home-based care for the sick and frail in SA. Under this tradition, healthcare needs are provided through home-based (i.e. home health) and community-based care (Bohman et al. 2009). If home-based care is chosen, implications for nursing include providing care truly at the *bedside*, thus creating greater roles for home care (home health; community), public health, and parish nurses in SA.

However, in response to global ageing, ‘as the lifespan increases, this familial responsibility to maintain home-based care will be harder and harder to sustain’ (Norstrand, Moon & Tran 2012:7), and hence more and more older South Africans will become institutionalised. As a result of urbanisation there is a transition from traditional home care to institutionalised care, as children and grandchildren caregivers relocate to cities (Bohman et al. 2009). Consequently, service-users of long-term care (i.e. permanent, continuous care of older adults in their later years) will grow substantially, raising the need for more long-term care globally. Hirschfeld (2009:105) affirms that chronic conditions such as HIV or AIDS and malaria, along with diseases of lifestyle, will require a larger utilisation of long-term care, even asserting that developing countries in sub-Saharan Africa, Latin America and the Eastern Mediterranean will experience increases of 300% – 400% in long-term care needs over the next decades.

Interestingly, nursing practice in the USA has begun a reverse migration, whereby an increasing amount of nursing care takes place in the home and community versus in institutional facilities. This is reflective of the ageing in place (remaining in one's home during the ageing life-course) philosophy of care. Another philosophy of care in the USA that piggybacks on the ageing in place movement is ‘hospital at home’, where skilled nursing care is provided in the home for older adults with chronic illnesses and some acute illness exacerbations. Hospitals are accessed only for acute illnesses that cannot be safely or effectively managed in the home. The goal is to keep older adults in their original environment as this promotes continuity of care, delirium reduction, personal security, autonomy, independence, and the right to self-determination. Even the smallest changes in or transitions to new environments can trigger the physical, functional and psychological decline cascade.

### Lesson 4: There is a need for gerontological nursing education in South Africa

Whilst in recent years the South African government has recommended a change in core content on older adult care in the educational curriculum, there remains a lack of gerontology in the SA nursing curriculum as attention is focused more on the larger public health problems at hand. From casual conversations with nursing students and faculty, emphasis on content which is specific to ageing or gerontology as presented in nursing school is minimal; nursing students receive a general nursing education in order to care for a broader patient population. This is slightly different from in the USA, where nursing students receive a general education delineated according to patient populations (e.g. geriatrics, paediatrics, psychiatric/mental health, etc.). Then nurses usually go on to work with and/or specialise in caring for a particular patient population; for example, nurses caring solely for older adults may specialise as gerontological nurses.

### Lesson 5: Holistic care is preferred and practised versus specialised care

Although nursing care in the USA is grounded in holism, whole-person care, and patient- and family-centred care, there is a focus on specialty care in order to provide the best care possible for specific health conditions. During an informal group conversation one South African nursing student pointed out that care in the USA is very specialised and sometimes isolated. I responded by acknowledging the presence of specialised care, but also emphasised our use of interdisciplinary healthcare teams.

After reflecting on this earlier conversation and observations, the South African student's assertion remains partially true. In contrast to the USA, the implementation of holistic care is different in SA; SA nursing uniquely emphasises and operationalises the true essence of holistic (wholistic) care. It was quite clear that a ‘village’ approach (i.e. interdisciplinary collaboration with family/friends/community) was much more at the fore in SA, in that the health of the community and the help of the community are intertwined. I was left with the affirmation that contextual needs and cultural practices determine how holistic care is defined and implemented.

## Discussion

Ageing in all countries is operationalised very differently. Some principles of care are applicable across the world, but it is important to be sensitive to and knowledgeable about the differences in care. Therefore nurse educators are charged with educating nursing students on global ageing and cultural sensitivity (Tweedie 2012). The keys to successful ageing in SA will depend largely upon the nursing profession and family caregivers. Many older adults, particularly women, are primary caregivers for their household (Schatz 2007). Thus community-based nursing care can assist older adults in not only caring for themselves but also for family members (Mtshali 2009).

Foundational principles of ageing in place are that older persons are able to maintain a sense of normalcy, purpose in life, safety, and functional health in their current home environment. Yet environmental factors and increases in persons becoming infected with HIV and AIDS at older ages, combined with shortages of nursing and healthcare supplies make ageing in place more complex in SA. In addition, advances in healthcare technology and pharmacological treatment for various health conditions will lead to a dramatic increase in the number of older adults. Consequently, in the coming years ageing in SA will be of higher priority and will require a new social and nursing infrastructure to meet this growing need.

Therefore, nursing students and nurses will need strategic education on how to care for an ageing society. However, one issue that may hinder advancement of gerontological nursing is a shortage of nurses. A steadily ageing population coupled with a shortage of nurses warrants a focus on nurse burn-out and retention rates. Accordingly national policy initiatives have begun to address the shortage of nurses and the needed transformation of nursing education, practice, and collaboration standards in SA (Daniels & Khanyile 2013; Jooste & Jasper 2012; Seekoe 2014).

Whilst infusing gerontology information into the curriculum may be a challenge, recommendations and resources are provided in the next section to assist in building gerontological nursing capacity in SA.

### Recommendations for nursing in South Africa

To better care for older adults in SA and prevent avoidable adverse events (Bateman 2012), dedicated gerontology and geriatric concepts and evidence-based practices must be incorporated into nursing education, particularly since gerontological nursing is not a core clinical specialisa­tion programme (Vasuthevan 2013:287−288). Therefore gerontological nursing education standards have been developed in order to care for older adults more effectively and improve patient outcomes (American Nurses Association 2010). As a global nursing community there are several ways to bolster gerontological nursing capacity and infuse education on best nursing practices for older adults in SA.

An unquenchable yearning for knowledge in SA was evidenced by many of the nursing students asking numerous questions related to health care and nursing in the USA. The strategies outlined below may help to advance the care of older adults in SA.

#### Collaboration and partnerships

- Collaboration between nursing schools in SA and nursing centres in the USA, such as National Hartford Center of Gerontological Nursing Excellence, the Hartford Institute of Geriatric Nursing, and the Donald W. Reynolds Center for Geriatric Nursing Excellence, to infuse gerontological nursing into nursing curricula.
- Develop nursing care of older adults’ special interest groups in SA.
- Expand existing gerontological organisations to include international chapters.
- Develop a gerontological-focused international service-learning experience and exchange programme to educate South African nursing students and current nurses on care of older people.
- Join forces with the IAGG and Association for Gerontology in Higher Education.
- Develop international service-learning programmes with organisations such as Nurses Without Borders.
- Donations: US schools of nursing could donate resources that are needed to care for older adults.

#### Education

- Partner with South African schools of nursing to develop a gerontological curriculum that includes a stand-alone gerontological course.
- Increase the number of visiting gerontological nurse scholars in SA and the USA.
- Use the book *The State of Nursing and Nursing Education in Africa: A Country-By-Country Review* (Vasuthevan 2013) as tool to understanding nursing educational needs in SA.
- Access education and practice resources dedicated to care of older adults (see Booker (2014) for a list of resources). A few examples include: National League for Nursing Advancing Care Excellence for Seniors (http://www.nln.org/facultyprograms/facultyresources/aces/index.htm), and Hartford Institute for Geriatric Nursing (http://hartfordign.org/).

#### Practice

- Enhance clinical opportunities to work with diverse older adults (including indigenous and immigrant populations) in each community, long-term care, and acute care settings.
- Ensure simulation scenarios integrate aspects of culture, geriatrics, and intergenerational health.
- US nurses and nursing education should ensure adherence to culturally and linguistically associated services (CLAS) standards.
- Research: Expand American and South African nursing research to involve a global audience in order to develop and promote evidence-based care of older adults worldwide.

## Conclusion

The world is ageing rapidly, and many developed and developing countries remain under-prepared to care for this high service-using population effectively. The South African service-learning experience was life-changing and unforgettable, and is one way to develop cross-cultural gerontological nursing capacity.

One of the greatest takeaways from the experience was a deeper understanding of what it means to meet the specific needs of a population through application of evidence-based gerontological practices whilst upholding personhood, dignity, and cultural traditions.

A difference in care practices does not mean that one way is right; instead, it reflects what is right for the needs of that unique people. In SA I discovered a renewed sense of self, a higher role as a nurse, and an enhanced gratitude to my African ancestors.

Ageing is not what the chronological or biological number does to you, but rather what you do with age (gerotranscendence). Thus our mission as gerontological nurses should be, as the United Nations Declaration of the Rights of the Elderly states: ‘To add life to the years that have been added to life.’
